# Effect of Complexity on Speech Sound Development: Evidence From Meta-Analysis Review of Treatment-Based Studies

**DOI:** 10.3389/fpsyg.2021.651900

**Published:** 2021-04-28

**Authors:** Akshay R. Maggu, René Kager, Carol K. S. To, Judy S. K. Kwan, Patrick C. M. Wong

**Affiliations:** ^1^Department of Linguistics and Modern Languages, The Chinese University of Hong Kong, Hong Kong, China; ^2^Department of Psychology and Neuroscience, Duke University, Durham, NC, United States; ^3^Brain and Mind Institute, The Chinese University of Hong Kong, Hong Kong, China; ^4^Department of Languages, Literature and Communication, Utrecht Institute of Linguistics OTS, Utrecht University, Utrecht, Netherlands; ^5^The Chinese University of Hong Kong – Utrecht University Joint Centre for Language, Mind and Brain, Hong Kong, China; ^6^Division of Speech and Hearing Sciences, The University of Hong Kong, Hong Kong, China

**Keywords:** complexity, language learnability, optimality theory, markedness hierarchy, speech sound disorders

## Abstract

In the current study, we aimed at understanding the effect of exposure to complex input on speech sound development, by conducting a systematic meta-analysis review of the existing treatment-based studies employing complex input in children with speech sound disorders. In the meta-analysis review, using a list of inclusion criteria, we narrowed 280 studies down to 12 studies. Data from these studies were extracted to calculate effect sizes that were plotted as forest plots to determine the efficacy of complexity-based treatment approaches. The outcome variables of interest were improvement on the treated and generalization to the untreated sounds. Meta-analysis revealed that the exposure to complex input not only promoted improvement in production of complex speech sounds (*d* = 1.08, CI = 0.98–1.19) but also facilitated the production of untreated simple speech sounds (*d* = 2.69, CI = 1.98–3.54). Overall, the current findings revealed that the exposure to complex input promotes acquisition of both complex and simple speech sounds. The current findings are in line with the models of language learnability. The current findings have implications in the treatment of speech sound disorders.

## Introduction

Speech sound development is fundamental to spoken language and is a subject of theoretical debates. Whether or not the exposure to complex input first promotes speech sound development, is an intriguing question in the field of speech and language research. Although the complexity-based perspective on speech sound acquisition suggests that exposure to complex/less stimulable/later-acquired input promotes speech sound development (Gierut et al., 1987, 1996; Powell et al., 1991; Morrisette et al., 2003), variability across the existing studies makes it difficult to interpret the effect of complexity on speech sound development. To contribute toward answering the question on whether or not starting with complex input is beneficial for speech sound development, in the current study, we reviewed the existing treatment-based studies grounded on complexity perspective and conducted a meta-analysis on their findings. Treatment-based studies provide us with an excellent opportunity to experimentally investigate the psychological reality of theories, by selectively manipulating the treatment variables (Barlow and Gierut, 1999) to observe effects on atypical phonological patterns in population with speech sound disorders (Ferrier and Davis, 1973; Blache and Parsons, 1980; Elbert et al., 1980; Blache et al., 1981).

Complexity of input could range from complexity due to linguistic factors, psycholinguistic structure, articulatory-phonetic factors, and conventional clinical factors (Gierut, 2001). The concept of linguistic complexity, more specifically, phonological complexity, stems from universal grammar, or innateness perspective (Jakobson, 1941; Wexler and Culicover, 1980; Wexler, 1982; Prince and Smolensky, 1993; Tesar and Smolensky, 1998; McCarthy, 2007). Complexity-based theories support that the introduction of more complex (more marked) structures in the therapy promotes the development of both complex (more marked) and simple (less marked) structures. This is mainly because marked or more complex structures expose a child to surface forms that cannot yet be generated by their internal grammar, triggering the improvement of other structures with an equivalent or lesser complexity leading to an overall change in their language system (Gierut et al., 1987, 1996; Powell et al., 1991; Tyler and Figurski, 1994; Morrisette et al., 2003; Thompson et al., 2003; Thompson and Shapiro, 2007). *Psycholinguistic complexity* is based on the characteristics of words that affect word recognition in perception and production. For example, high frequency words are known to be more complex at a sublexical level as compared to words with low frequency (Gierut, 2001). As a result, use of high frequency leads to greater generalization and change in the sound system than the words with low frequency (Gierut et al., 1999). *Articulatory-phonetic complexity* refers to complexity of speech sounds based on the ease of pronunciation and perception. For example, non-stimulable sounds could be defined as more complex as compared to stimulable sounds. Treatment with non-stimulable sounds leads to more generalization to both stimulable and non-stimulable sounds while treatment with stimulable sounds only generalizes to treated stimulable sounds but not non-stimulable sounds (Powell et al., 1991). Complexity due to *conventional clinical factors* includes complexity due to clinical aspects, methodological strategies, and/or techniques. For example, a sound which is a consistent error is a more complex input than a sound which is an inconsistent error, a sound that is later-acquired is more complex than an early-acquired sound, and pairing two new (i.e., unacquired) sounds in a minimal pair becomes harder to learn than pairing a new sound with an old sound (i.e., acquired vs. unacquired). Gierut (2001) found that by using stimuli that are more complex, as defined above, one can achieve better generalization learning as compared to using simple stimuli.

### Complexity Theories-Based Treatment

A series of studies (Gierut et al., 1987, 1996; Powell et al., 1991; Tyler and Figurski, 1994; Morrisette et al., 2003) have been conducted using linguistic complexity in the treatment of children with speech sound disorders. For example, studies that have employed markedness hierarchies of fricatives > stops (Dinnsen and Elbert, 1984) and consonant clusters > affricates (Dinnsen, 2008), revealed that exposure to more marked or more complex speech sound [e.g., fricatives in Dinnsen and Elbert (1984)] led to improvement in complex speech sound as well as generalization to untreated simple speech sound [e.g., stops in Dinnsen and Elbert (1984)]. On the other hand, exposure to less marked or simple speech sound led to improvement on simple speech sound but did not promote generalization of untreated complex speech sound. Along with the studies based on linguistic complexity, there are studies (Gierut et al., 1987; Gierut, 1990, 1991, 1992; Williams, 2000; Peach and Wong, 2004) that use complexity based on psycholinguistic, acoustic-phonetic, and methodological or technical factors. For example, Gierut (1992) found that training with 2-new phonemes (complex) led to greater generalization than training with 1-new phoneme (simple). Overall, findings from these studies reveal an enhanced improvement in speech sound production with the use of complexity.

### The Current Study

Though there is evidence favoring complexity-based (Dinnsen and Elbert, 1984; Gierut et al., 1987, 1996; Powell et al., 1991; Tyler and Figurski, 1994; Barlow and Gierut, 1999; Morrisette et al., 2003; Gierut, 2008) procedures, there is a considerable variability in the magnitude of effects across the studies that make it difficult to understand the efficacy of the complexity approach. One way to understand the efficacy of the complexity approach is by conducting a meta-analysis in which the data from the existing literature are extracted, processed, and plotted together to conduct a quantitative systematic review. Previously, review studies (Law et al., 2003, 2004; Nelson et al., 2006; Baker and McLeod, 2011; Furlong et al., 2021) have discussed different techniques for the treatment of speech sound disorders. However, these review studies were conducted with a pure clinical perspective to guide the speech language pathologists (SLPs) in their clinical practice and were not aimed at addressing the theoretical question on whether or not complexity facilitates speech sound acquisition/development.

In the current research, we tried to bring a more conclusive understanding to this theoretical question by conducting a meta-analysis of the data extracted from the existing complexity-based literature on the treatment of speech sound disorders. In the current study, we included studies with single-case-experimental-design (SCED), that account for much of the research (29.6%) on speech sound disorders (Baker and McLeod, 2011) but ignored in the previous clinical-based reviews (Law et al., 2003, 2004; Nelson et al., 2006; Furlong et al., 2021). SCEDs play a key role in treatment-based studies for the following reasons: (1) SCEDs work well with a heterogeneous population. Children with speech sound disorders often display phonological profiles which are different from one another, quantitatively and/or qualitatively; (2) As participants are evaluated at multiple time-points in the baseline as well as treatment conditions, each participant serves as his/her own experimental control; (3) SCEDs can account for maturation thereby strengthening the internal validity of the treatment-based studies; (4) SCED is more relevant to clinical practice in communication disorders as it examines changes within a patient. In addition, SCED data from many subjects can be combined to form groups as well. Additionally, Gierut (2008) argues that data collected with multiple baseline single subject designs yield many more data points as compared to group-level study. Given the advantages of SCEDs, the current meta-analysis focused on this important but largely ignored volume of research on the treatment of speech sound disorders.

In the current study, along with measuring the outcome on treated sounds, we also examined the generalization of treatment to other untreated sounds. As generalization to other untreated sounds reflects widespread changes in the phonological system, it forms an important measure of treatment efficacy (Gierut, 1998). The scores on the outcome measures of all the selected studies were converted to effect size [i.e., Cohen's *d* or Standardized Mean Difference (SMD)]. We predicted that if the overall effect size, derived from the combination of effect sizes from the complexity-based treatment procedures extracted via literature search, turned out to be significant, it would imply that exposure to complex stimuli first contributes toward speech sound development. On the contrary, if the overall effect size turned out to be non-significant, it would imply that exposure to complexity first does not contribute toward speech sound development.

## Methods

### Identification of Studies

Before we carried out any searches, we developed inclusion criteria for studies based on the study design, types of intervention, age of participants, and outcomes (Table 1). The relevant literature was obtained by searching for studies in literature databases consisting of Google Scholar, Campbell Collaboration, Cochrane Database of Systematic Reviews, EMBASE, PsychINFO, and MEDLINE.

**Table 1:** Inclusion criteria.

**Design** Participants were treated in a Single-Case-Experimental-Design (SCED) that includes single or multiple baselines AB, ABA designs. All other research designs were excluded
**Types of intervention** Studies related to complexity-based approaches were included
**Participants** Preschoolers and school-age children diagnosed with speech sound disorders
**Outcomes** Post-therapy scores on phonology or articulation testing


**Figure 1:**
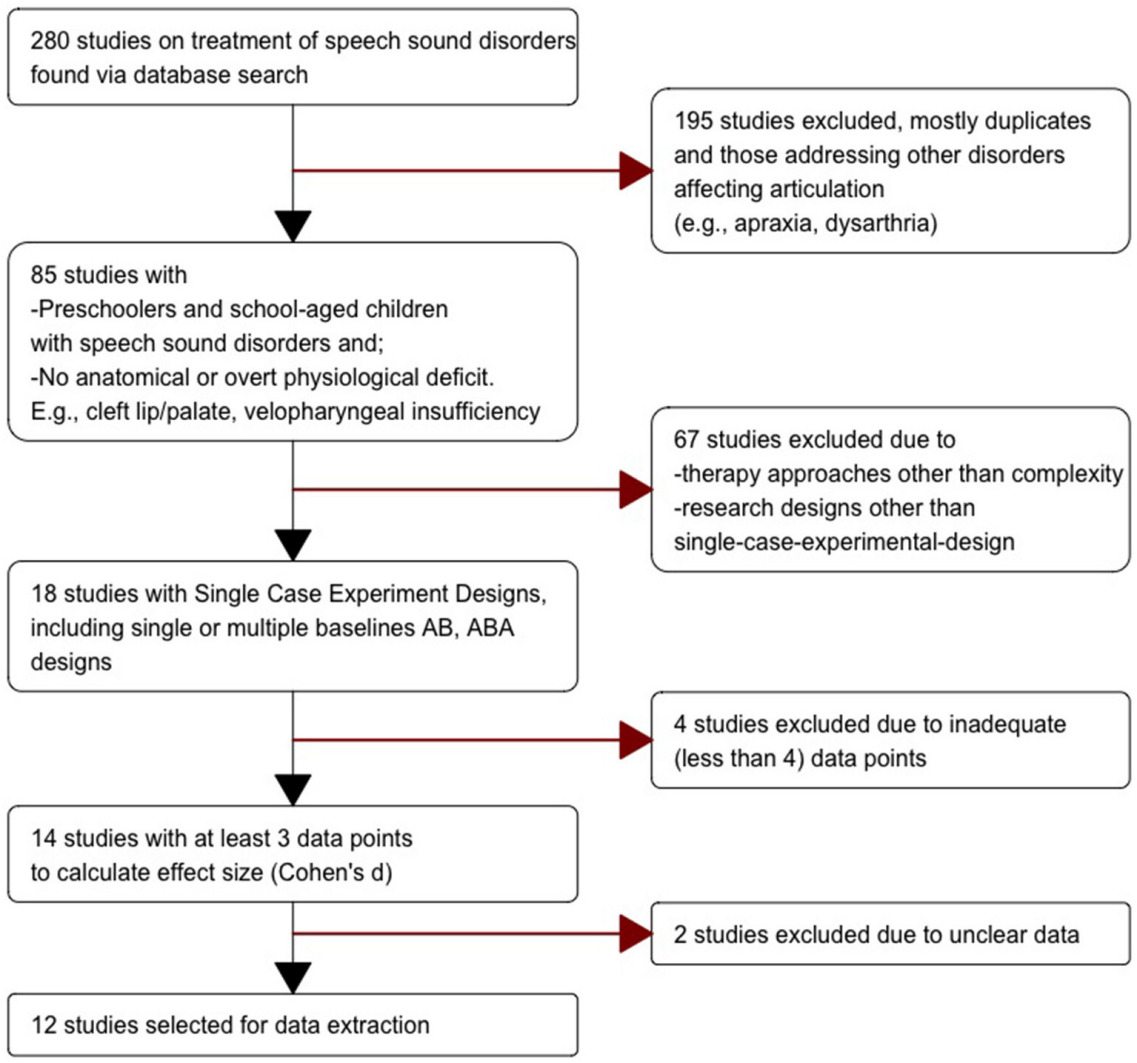
PRISMA chart showing the process of study identification for meta-analysis.

The keywords mentioned below, and/or their combinations were used to search for the relevant literature: children, school, articulation, clinical, phonology, Optimality Theory, complexity, differential, treatment, minimal pair, early/late-acquired, stimulable/non-stimulable, auditory, and training. The inclusion criteria (Table 1) were applied in a series of five hierarchical steps starting with a broad search criterion in step 1 with 280 studies, narrowing it down to step 5 with 12 studies (Figure 1). The current study adhered to the reporting standards of PRISMA (Moher et al., 2009) and SCRIBE (Tate et al., 2016).

### Coding

Studies that met the inclusion criteria (Table 1) were coded for research design, participants, types of intervention, and outcome (see Supplementary Table 1). All the 12 selected studies were independently coded by the first author and the second rater. Agreement of ~87% was established between the coders and opinion from the last author was sought for the disagreements. The second rater and the last author were blinded to the quantitative results at the time the classification was made.

### Participants

Studies with preschoolers and school-age participants were considered in this meta-analysis review. The participants from all these studies were native English speakers ranging from 3;5 to 6;10 years of age. They all had hearing abilities within normative limits and normal oral and speech motor abilities.

### Interventions

Studies were coded for their research designs, mode, and total duration of service delivery. Some studies were given multiple codes as they studied the effect of complexity on treated complex speech sounds and untreated simple speech sounds.

### Outcomes

So as to obtain homogeneity, for the purposes of meta-analysis, it was ensured that all studies focused on similar outcome measures. For example, scores on post-therapy measures. Outcomes of interventions for treated and untreated (whenever available) speech sounds were coded.

### Extraction of Data

In order to calculate the effect sizes, data were either extracted from the tables in the studies or they were retrieved from the graphs in the studies using a pencil and a ruler (Beeson and Robey, 2006). These data were converted to percentage values to further calculate the effect sizes.

### Calculation of Effect Size

Effect size (i.e., Cohen's *d* or SMD) was calculated following the existing recommendations (Beeson and Robey, 2006). The data points were considered both for pre- and post-therapy. Usually Cohen's *d* is calculated as

$$\begin{array}{l} \text{d}=\left({\bar{x}}_{\text{A}2}-{\bar{x}}_{\text{A}1}\right)/SD_{\text{A}1} \end{array}$$

In the above formula, “d” refers to the effect size, “${\bar{x}}_{\text{A}2}$” refers to the mean of post-therapy data points, “${\bar{x}}_{\text{A}1}$” refers to the mean of pre-therapy data points, and SD_A1_ refers to the standard deviation obtained with the pre-therapy data points. However, as a few studies had zero-variance values in the pre-therapy baseline, it was not possible to calculate the standard deviation (SD) for the pre-therapy condition. Instead, a pooled SD was derived by combining SDs of both pre- and post-therapy (Beeson and Robey, 2006). The effect size calculated using the pooled SD was calculated as d_2_ (Busk and Serlin, 1992).

$$\begin{array}{l} d_{2}=\left({\bar{x}}_{\text{A}2}-{\bar{x}}_{\text{A}1}\right)/SD\text{pooled} \end{array}$$

where A_2_ is post-therapy evaluation and A_1_ is pre-therapy evaluation.

Effect sizes were weighted for the number of observations in the pre- and post-therapy assessments. Further, effect sizes from each study were weighted for the number of subjects to obtain a *summary* (overall) effect size (Beeson and Robey, 2006).

## Results

Supplementary Table 1 provides a summary of the studies included in the current meta-analysis. The therapy procedures were criterion-dependent (targeting 75–90%) and/or duration-dependent (≤ 20 sessions). Service delivery ranged from home- to school- to clinic-based therapy. Effect size was calculated as standardized mean difference (SMD), also known as Cohen's *d*, obtained by subtracting pretest means from post-test means relative to the variability observed in the non-treatment period (pre- and post-therapy). Effect sizes were plotted on forest plots with confidence intervals on either side so that comparison across studies could be done in an efficient manner.

### Analyses

The aim of the current study was to extract and analyze the data from the literature pertaining to the use of complexity approach and thus, provide a quantitative understanding toward the effect of complexity on speech sound development. The effectiveness of the approach was analyzed on the treated as well as untreated speech sound categories. Out of the 12 selected studies (*N* = 50) that studied the effect of complexity on treated speech sounds, 5 studies (*n* = 23) examined the generalization to untreated speech sounds (Table 2).

**Table 2:** Distribution of the included studies across the outcome variables of treated complex speech sounds and untreated simple speech sounds.

**References**	**Effect of complexity-based**	
	**approach on**	
	**Treated complex** **speech sounds** **(*n* = 12)**	**Untreated simple** **speech sounds** **(*n* = 5)**
Williams (1991)	+	
Williams (2000)	+	+
Powell and Elbert (1984)	+	+
Gierut and Champion (1999)	+	
Gierut et al. (1996)	+	+
Gierut et al. (1987)	+	+
Powell et al. (1998)	+	
Gierut (1992)	+	
Gierut (1991)	+	
Gierut and Neumann (1992)	+	
Miccio and Ingrisano (2000)	+	+
Gierut (1990)	+	

**Figure 2:**
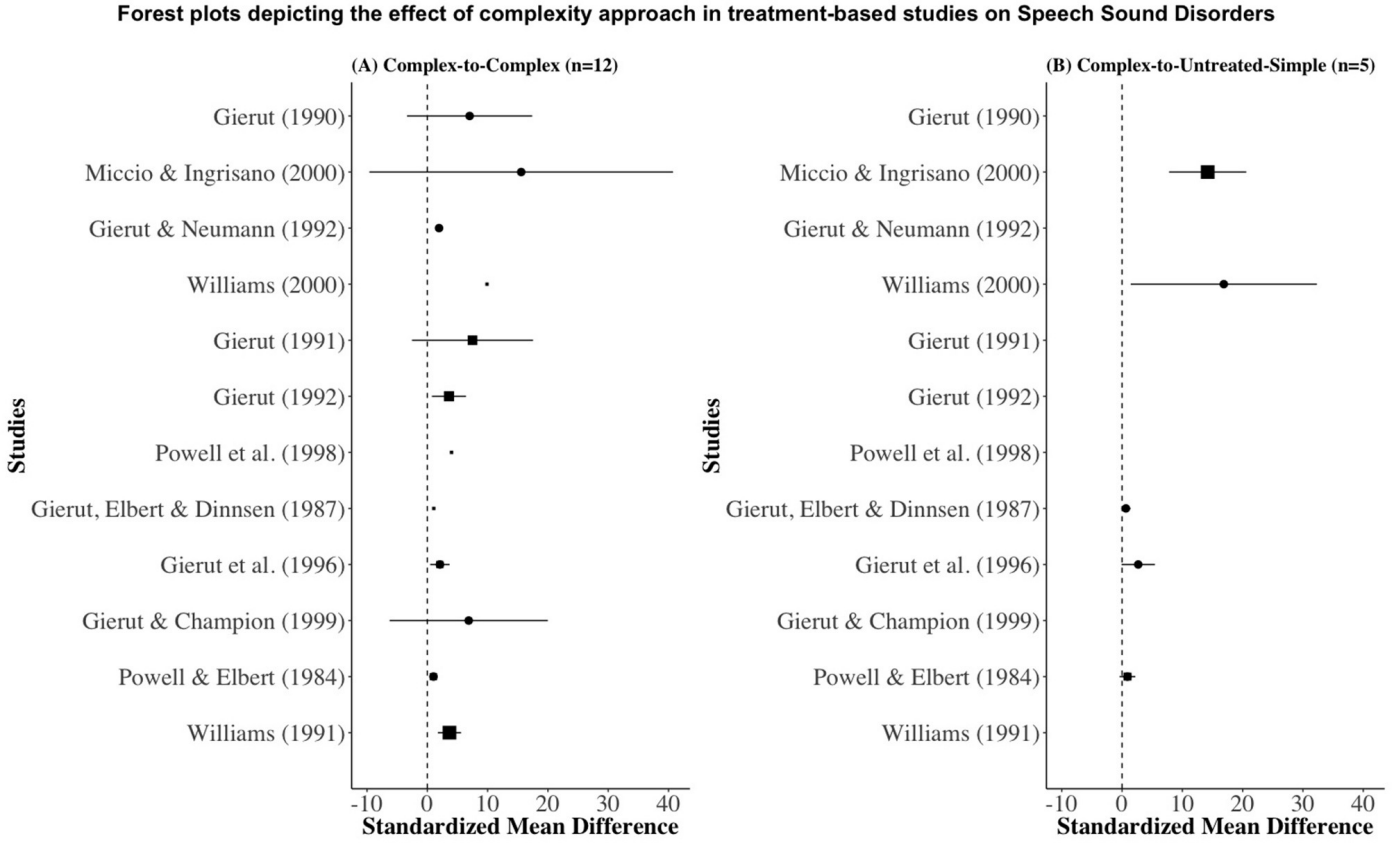
Forest plots depicting the effect of complexity approach in treatment of speech sound disorders. **(A)** Effect of complexity approach on treated complex sounds; **(B)** Effect of complexity approach on untreated simple sounds.

With the data extracted from the selected studies (Table 2), forest plots were constructed to depict the effect sizes (Figure 2). In these forest plots, abscissa represents SMD and ordinate contains reference of the studies included in the current meta-analysis. The solid square with lines emerging from either end are effect size with confidence intervals (C.I.). The width of the solid square reflects the weight contributed by the respective studies toward the overall effect size.

**Figure 3:**
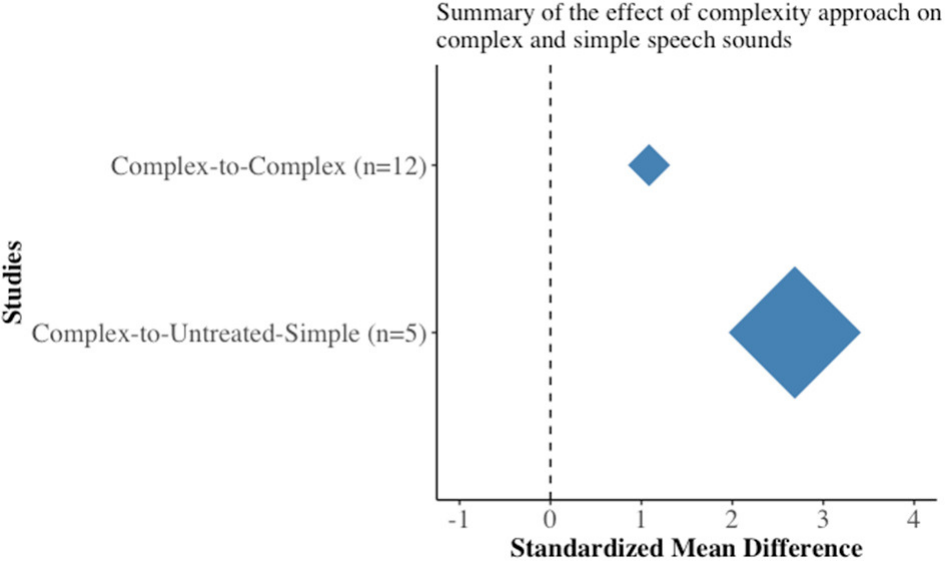
Diamond plot depicting the summary (weighted) effect sizes on the treated complex and untreated simple speech sounds.

To gain a better appreciation on the overall performance of complexity-based approach on treated complex speech sounds and untreated simple speech sounds, weighted *summary* (overall) effect sizes were plotted separately in a diamond plot (Figure 3). The complexity-based approach not only led to an improvement in treated complex speech sounds (*d* = 1.08, *n* = 50, CI = 0.98–1.18) but also led to an improvement in the production of untreated simple speech sounds (*d* = 2.69, *n* = 23, CI = 1.98–3.54).

## Discussion

The current study aimed at understanding the influence of complex input on speech sound development. More specifically, we investigated this by conducting a systematic quantitative review of literature on complexity-based approaches of treatment of speech sound disorders. Based on our meta-analysis, we found that the complexity-based approach led to an improvement in treated complex speech stimuli and extended generalization to untreated simple speech stimuli.

Although the literature related to treatment of speech sound disorders has been reviewed from time to time (Law et al., 2003, 2004; Nelson et al., 2006; Baker and McLeod, 2011; Furlong et al., 2021), the current systematic review is different from the previous review studies in the following aspects: First, the current meta-analysis includes the studies with SCEDs which are one of the most used designs in phonological treatment-based studies (Baker and McLeod, 2011) but were largely ignored in the previous reviews (Law et al., 2004; Nelson et al., 2006). Second, the current review focused on a specific question on the effect of complexity approach in the treatment of speech sound disorders while the previous reviews (Gierut, 1998; Baker and McLeod, 2011) had a broad focus on the effects of intervention. Third, by examining the effect of complexity-based approach, the current review contributed toward answering the theoretical question on the effect of complex input on speech sound development.

The current findings are in agreement with the findings of Gierut and other researchers (Gierut et al., 1987; Powell et al., 1991; Tyler and Figurski, 1994; Morrisette et al., 2003) who suggest the use of a complex set of stimuli for treatment of children with speech sound disorders and are consistent with the models of language learnability (Wexler and Culicover, 1980; Wexler, 1982). From the current findings, it seems that complex input is more efficacious in triggering and promoting the development of both complex and simple speech sounds in a rule-governed manner. A plausible explanation for the findings of the current review that supports the notion of innateness, especially in the context of linguistic complexity, could be provided via demotion or differential promotion of markedness constraints resulting from the introduction of complex stimuli. Constraint demotion or differential promotion can only occur when complex stimuli are introduced. For example, in Lleó and Prinz (1996), when a cluster (more marked) was used for training, there was an emergence of correct productions of both clusters and affricates by constraint demotion of markedness constraints of both clusters and affricates. On the contrary, if the affricates (less marked) were used as stimuli, it merely led to the development of affricates by demotion of its markedness constraint beyond the faithfulness constraint. However, it did not promote clusters, mainly because the markedness constraints of clusters still remained higher ranked. In other words, when a complex stimulus is used, it maps on to the complex innate linguistic structures to promote the development of both complex and simpler speech sounds.

Along with theoretical implications, the current research also has clinical implications. Speech sound disorders, being one of the most prevalent child language disorders, constitute a major portion of caseloads of speech language pathologists (SLPs) dealing with pediatric cases (Baker and McLeod, 2004; Broomfield and Dodd, 2004; Mullen and Schooling, 2010). According to the National Institute of Deafness Other Communication Disorders (1994), prevalence of speech sound disorders ranges from 3 to 13% in the United States. Speech sound disorders affect about 10% of the pre-school and school-age children and constitutes a major portion of caseloads of SLPs rendering services at school (National Institute of Deafness Other Communication Disorders, 1994). Speech sound disorders can be comorbid with primary language impairment and learning disability and these can have a profound impact on a child's academic skills including reading, writing, spelling and mathematics (King et al., 1982; Shriberg and Kwiatkowski, 1985; Catts and Kamhi, 1986; Hoffman and Norris, 1989; Hoffman, 1990; Lewis and Freebairn, 1992; Webster and Plante, 1992; Catts, 1993; Bird et al., 1995; Clarke-Klein and Hodson, 1995; Ingvalson et al., 2015). Children with speech sound disorders usually do not attain similar educational and employment level as their typically developing peers (Felsenfeld et al., 1994; Dinnsen, 2008). Given the high incidence and lifelong effects of childhood speech sound disorders, early identification and intervention, especially for children in their pre- and primary schools, is warranted. A large number of different intervention approaches exist for speech sound disorders (Gierut, 1998; Baker and McLeod, 2011). Given the heavy caseload on the practicing pediatric SLPs, they have limited time to review all the relevant evidence for maximizing the effectiveness of the treatment they provide. The current meta-analysis can provide them with an empirical basis to employ complexity-based techniques in their clinical practice that not only improve the treated speech sounds but also generalize to untreated speech sounds of lesser complexity.

### Limitations of the Current Study

There are some limitations to the current research that will be discussed in this subsection. First, all the included studies were not homogenous in terms of their study design. Seven studies used multiple baseline AB designs, one study used single baseline AB, one study used multiple baseline ABA, and three studies used single baseline ABA design. As a result, the number of data points available for the calculation of effect sizes varied across studies. This problem was circumvented by calculating weighted average across the data points. Second, the studies varied in terms of whether blinding was used or not. Two of the 12 included studies had blinding while others did not. Even though we tried to establish an optimal inclusion criteria to maintain specificity of the included studies, in an ideal world, one might want to maintain homogeneity in all aspects.

### Validity of the Meta-Analysis

**Figure 4:**
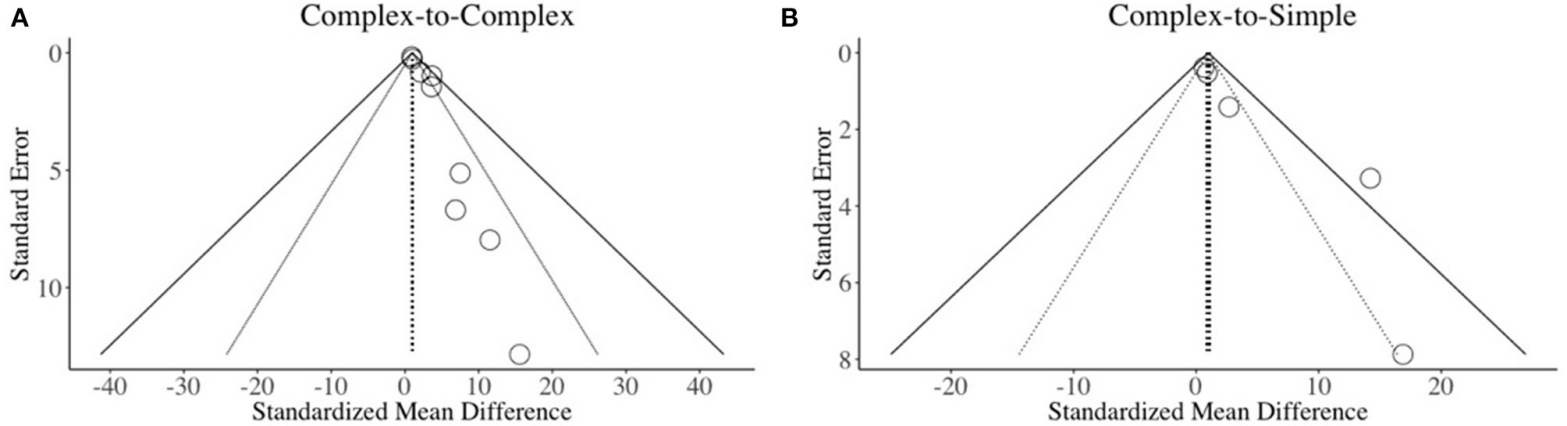
Funnel plots of different conditions. Dotted lines (·····) represent 95% CI while striped lines (–) represent 99% CI. Open circles (o) represent effect sizes plotted against standard error. **(A)** Complex-to-Complex: Effect of complex stimuli on treated complex speech sounds; **(B)** Complex-to-Simple: Effect of complex stimuli on untreated simple speech sounds.

There are at least two factors that can affect the validity of meta-analysis data: (1) Quality of studies: It is possible that meta-analysis results could be affected by the quality of studies included; (2) Selection bias: This could be caused by the inclusion of studies with big effect sizes while selectively ignoring the studies with low or negative effect sizes. This is also known as *bottom drawer* effect (Law et al., 2004). In order to evaluate the likelihood of publication bias (if any), we constructed funnel plots (Figure 4) as a function of standard errors and effect sizes of the studies distributed across the four categories of interest: (A) Complex-to-Complex: Effect of using complex stimuli on treated complex sounds (Figure 4A); (B) Complex-to-Simple: Effect of complex stimuli on untreated simple sounds (Figure 4B). Ideally, if the effect sizes are distributed on either side of the average effect size, the meta-analysis is said to be free from publication bias. From the funnel plots of the data extracted in the current review, we found that there were studies on either side (Figure 4) that confirm our study selection was free from publication bias. However, our data do not depict an absolute symmetrical distribution which could be due to main two reasons: (1) *Small sample size:* The studies that were included in these analyses had a sample as small as 1 in a single subject design; (2) *Treatment-related improvement:* The current review focused on treatment studies where subjects respond to treatment even though to a minimal degree.

### Future Directions

Speech language pathology as a profession is relatively young, and it has a shorter research tradition compared to other disciplines such as medicine (Dodd, 2008). Thus, it is unsurprising to find it lacking in the highest level of evidence and accumulation of case studies in the available literature. However, in order to gain more confidence in the treatment-related outcomes, future studies should employ randomized controlled trials that are considered the highest level of evidence by ASHA (Robey, 2004) and the Oxford Centre for Evidence-based Medicine - Levels of Evidence (2009). In addition, to make more conclusive remarks on the effect of complexity-based approaches, studies using a variety of speech sounds across different languages, different modes of service delivery, and subtypes of speech sound disorders should be conducted.

## Data Availability Statement

The original contributions generated for the study are included in the article/Supplementary Material, further inquiries can be directed to the corresponding authors.

## Author Contributions

AM, RK, CT, and PCMW designed the study and wrote the manuscript. AM and JK collected, compiled, and analyzed the data. All authors contributed to the article and approved the submitted version.

## Conflict of Interest

PCMW is the founder of a startup company supported by a Hong Kong SAR government tech-company startup scheme for universities. The remaining authors declare that the research was conducted in the absence of any commercial or financial relationships that could be construed as a potential conflict of interest.
