# Supplementary material for: Effect of Complexity on Speech Sound Development: Evidence From Meta-Analysis Review of Treatment-Based Studies
Source: Front Psychol. 2021 Apr 28;12:651900. doi: 10.3389/fpsyg.2021.651900 (PMC8113766; doi:10.3389/fpsyg.2021.651900)
Supplement: Supplementary file 1 [file Table_1.docx]

# Supplementary Information

Supplementary Table 1. Summary of studies coded for demographics, site of service delivery, study duration and research designs

| **Reference** | **Research Design** | **Participant number and age (in parenthesis)** | **Site of service delivery** | **Study duration** | **Summary of findings** | **Type of complexity**  **involved** |
| --- | --- | --- | --- | --- | --- | --- |
| Williams (1991) | Multiple-baseline AB design | n = 9 (3;8 - 5;9) | Not clear | 70% accuracy or maximum of 20 sessions | \| Found that the "least knowledge"  (complex) sounds  (clusters: /st/, /tr/) led to  improvement in 8 out of 9  subjects \| \| --- \| \|  \| | Conventional-clinical |
| Williams (2000) | ABA | n = 1 (3;5) | Clinic | 15 sessions | \| 1.Multiple opposition treatment  (complex paradigm) with /w/  against other sounds in minimal  pairs led to improvement in  both treated (/w/) and untreated  sounds (/s/, /sh) \| \| --- \| \| 2.Simple paradigm stimuli with  /w/-only sounds led to  improvement of /w/ but did not  generalize to untreated sounds  (/s/, /sh/) \| | Psycholinguistic |
| Powell and Elbert (1984) | Multiple-baseline AB design | n = 6 (4;4-6;3) | Clinic | 90% accuracy | 1. Training stop+liquid clusters (simple) improved treated stop+liquid but did not generalize to fricative+liquid (complex) 2. Training fricative+liquid (complex) improved treated fricative+liquid and generalized to untreated stop+liquid (simple) | Linguistic |
| Gierut and Champion (1999) | Multiple-baseline ABA design | n = 2 (4;0-4;8) | Clinic | 75% accuracy or maximum of 7 sessions | Investigated s-->θ-->f chain shift pattern and treated /s/ sound as it was on the end of the chain (most marked) and found significant improvement. | Linguistic |
| Gierut et al. (1996) | Multiple-baseline AB design | n = 9 (3;5-5;6) | Not clear | 75% accuracy of 2 consecutive sessions | 1. Treatment of later-acquired (/r/, /s/, /θ/) sounds led to more changes when compared to treatment with early-acquired sounds (/k/, /g/) 2. Treatment of more marked sounds (/s/) generalized more to untreated lesser marked sounds (other fricatives, stops) | Linguistic |
| Gierut et al. (1987) | Multiple-baseline AB design | n = 6 (3;7-4;6) | Clinic | 90% accuracy of two consecutive 30 minute sessions | 1. The subjects 1, 2 and 3 were treated with aspects labelled "most" knowledge (simple stimuli) and subjects 4, 5 and 6 were treated with aspects labelled as "least" knowledge (complex stimuli).Subjects treated with complex stimuli showed more improvement than those treated with simple stimuli | Conventional-clinical |
| Powell et al. (1998) | Multiple-baseline AB design | n = 6 (3;6-6;10) | Not clear | Maximum of 20 sessions | Overall, conceptual (phonological) training with /s/ (complex) led to more improvement as compared to treatment with simple motoric training of /s/ | Conventional-clinical |
| Gierut (1992) | Multiple-baseline AB design | n = 4 mean=3;10 | Clinic | 90% accuracy or maximum of 12 sessions | \| Investigated 2-new phoneme  (complex) vs 1-new phoneme  Strategy (simple) and found that  2-new phoneme strategy was  more successful \| \| --- \| \|  \| | Conventional-clinical |
| Gierut (1991) | Multiple-baseline AB design | n = 2 (4-5) | Home | 75% accuracy of 2 consecutive sessions | Homonymous minimal pairs (simple) vs unknown set (complex) strategy: Found that treatment with unknown set led to more improvement than homonymous pairs | Conventional-clinical |
| Gierut and Neumann (1992) | AB | n = 1 (4;8) | Clinic | 75% accuracy of 2 consecutive sessions then 90% accuracy over 3 consecutive sessions | \| 1. Treated for homonymous   condition: /s, t/ \| \| --- \| \| 1. Treated for non-homonymous   condition: /sh,Θ/  Found more improvement with  homonymous (complex)  than non-homonymous  condition (simple) \| | Conventional-clinical |
| Miccio and Ingrisano (2000) | ABA | n = 1 (5;3) | School | 90% accuracy of 3 consecutive sessions | Complex stimuli (/v/, /z/) led to improvement in treated complex sounds (/v/, /z/) and generalized to untreated simple speech sounds (/f/, /θ/, /s/) | Linguistic |
| Gierut (1990) | ABA | n = 3 (4) | Clinic | 75% accuracy of 2 consecutive sessions | Maximal opposition (simple; /g-m/) versus minimal opposition (complex; e.g. /s-t/,) compared. Maximal opposition led to greatest success | Psycholinguistic |
